# Water intake among Ghanaian youth aged 15–34 years: quantitative and qualitative evidence

**DOI:** 10.1186/s41043-018-0135-3

**Published:** 2018-02-05

**Authors:** Phidelia Theresa Doegah, Acheampong Yaw Amoateng

**Affiliations:** 1 Institute of Health Research, University of Health and Allied Sciences, Ho, Ghana; 2 Research Focus Area, Faculty of Human and Social Sciences, North-West University (Mafikeng Campus), Mahikeng, South Africa

**Keywords:** Quantitative, Qualitative, Youth, Water intake, Ghana

## Abstract

**Background:**

The prevalence of obesity is on the increase in Ghana, and the intake of sugar-sweetened beverages may be a determinant. The aim of this study is to use quantitative and qualitative data to investigate water intake among Ghanaian youth 15–34 years old.

**Methods:**

The 2008 Ghana Demographic and Health Survey data is used to investigate the effects of socio-demographic factors on water intake behaviours of a sample of 2771 male and 2806 female youth aged 15–34 years old in Ghana. Additionally, data from focus group discussions are used to examine perceptions with regard to water intake. In terms of the analysis, the quantitative bit of the data utilised Stata software and the qualitative data used the Atlas Ti software. Percentages, means, standard deviations, *t* test, one-way ANOVA, Tukey’s post hoc test and Poisson regression were used for the quantitative analysis while the qualitative data was analysed thematically.

**Results:**

Quantitative results found that age (IRR = 1.112, 95% CI 1.070–1.156) and region of residence (IRR = 0.855, 95% CI 0.780–0.937; IRR = 0.910, 95% CI 0.834–0.993) were important predictors of water intake in males, while age (IRR = 1.103, 95% CI 1.054–1.153), region of residence (IRR = 0.907, 95% CI 0.844–0.975; IRR = 1.258, 95% CI 1.130–1.400), ethnicity (IRR = 0.919, 95% CI 0.834–1.013) and marital status (IRR = 1.051, 95% CI 0.999–1.106) were found to be important predictors of water intake among females. From the focus group discussion, accessibility and physiological factors were mentioned as issues hampering adequate water intake.

**Conclusion:**

The similarities and differences between males and females should sensitise policy makers to the need for more gender-specific interventions to encourage water intake for the purposes of preventing non-communicable diseases. Moreover, intervention(s) to promote water intake should address issues of accessibility, physiological factors, weather and weight management.

## Background

According to the World Health Organization (WHO) [1], there has been a shift in the major causes of death from infectious to modifiable non-communicable diseases (NCDs). The prevalence of NCDs has been found to be high among the youth of sub-Saharan Africa [2]. For instance, hypertension prevalence of 22.3 and 6% was recorded in South Africa and Ghana, respectively [3, 4]. These lifestyle diseases usually emerge in middle age after a long exposure to such unhealthy lifestyle behaviours as the use of tobacco and alcohol, lack of regular physical activity and consumption of diets rich in saturated fats, sugars and salt [5, 2]. Among these unhealthy lifestyles by the youth is the regular intake of sugar-sweetened beverages which are associated with negative health risks, including obesity [6].

In Ghana, NCDs such as stroke, hypertension and type 2 diabetes are now among the top ten in-patient causes of death in the country [7], while obesity has been found to be a risk factor for these NCDs. According to the 1993–2003 Ghana Demographic and Health Survey (GDHS), there has been an increasing prevalence of female obesity (10 to 25.3%) in the country [8–10]. It is on the basis of these facts about the unhealthy lifestyles of the youth that the Ministry of Health (MoH) [11] in its Regenerative Health and Nutrition Programme (RHNP) recommends a daily consumption of at least eight glasses of potable water.

Water is calorie-free and therefore poses no danger to health, and as such, its consumption is expected to protect individuals from obesity and NCDs [11]. Tate et al. [6] observed an association between weight reduction and the replacement of sugar beverages with water, while other studies have found that the consumption of water is affected by such socio-demographic factors as age, education, ethnicity and region of residence [12–14].

For example, Park et al. [14], in a study in the USA, found that persons aged 15 years or younger drank less water, while Goodman et al. [13], using a nationally representative data in the USA, also found that respondents aged 18–34 years drank more water compared to those aged 55 or older. Also, Drewnowski et al. [12] used data from the National Health and Nutrition Examination Survey (NHANES; 2005–2010) and found that older adults (≥ 71) consumed lesser amount of water compared to younger persons (20–50 years). Because individuals aged 25–34 years may be more health conscious, in the present study, they are expected to consume more water than those aged between 15 and 24 years.

In terms of ethnicity and water intake, while some studies found no association [12, 14], Goodman et al. [13] in the USA showed a low probability for subjects belonging to ‘other’ ethnic groups in drinking adequate amount of water compared to whites. Because of their location in a warmer clime in Ghana, in the present study, respondents who are of the Mole-Dagbani group will be more likely to consume more water than the other ethnic groups.

As far as region is concerned, Goodman et al. [13] in a study in the USA found a statistically significant association between region of respondents’ residence and water intake. Specifically, they found that respondents in the Northeast region had a lower probability of consuming adequate water compared to their counterparts in the South. The present study hypothesises that respondents in the Northern, Upper East and Upper West regions will consume more water than residents in other regions of the country because of their location in warmer climes in the country.

### The present study

As the above review has shown, while a lot of studies on the relationship between socio-demographic factors and water intake have been done in other contexts, to our knowledge, no such studies have been conducted using Ghanaian samples. The current study seeks to fill this void in the literature by examining the association between selected socio-demographic factors and the consumption of potable water. Specifically, the present study uses the 2008 Ghana Demographic and Health Survey data and eight focus group discussions (FGDs) to examine the consumption of potable water among Ghanaian youth aged 15–34 years.

## Methods

The data for the study was derived from two main sources: first, the 2008 Ghana Demographic and Health Survey (GDHS) and second, eight focus group discussions (FGDs). The GDHS [15] is based on a sample of 12,323 selected households nationwide. It employed a two-stage sampling design whereby the first stage involved the selection of sample points or clusters from an updated master sampling frame constructed from the 2000 Ghana Population and Housing Census.

A total of 412 clusters were selected from the master sampling frame using systematic sampling with probability proportional to size (*PPS*). The second stage involved the systematic sampling of 30 households from each cluster. In terms of the data collection, three questionnaires were used, namely, (1) household questionnaire, (2) women questionnaire and (3) men questionnaire. The men and women questionnaires were administered to those in the 15–59 and 15–49 age brackets respectively. The quantitative part of the present study which examines the socio-demographic (age, education, religion, ethnicity, marital status, place and region of residence) determinants of water consumption by the youth in Ghana uses the GDHS.

To examine youth’s perceptions regarding water consumption, data generated from the eight focus group discussions were used. The FGDs were conducted within two urbanised towns in the Greater Accra and Volta regions respectively between September (2015) and January (2016). Participants were divided into two groups 15–24 years and 25–34 years, and each age group had two groups comprising males and females. Participants for the FGDs were sampled until saturation was reached.

For each age group and sex, there were two FGDs, yielding a total of eight FGDs. They were structured around these themes: the perceptions regarding water consumption and perceived barriers to adequate water consumption. Interviews were conducted in the Ga, Ewe and English languages respectively. Ethical approval (NWU-00224-15-A9) from the Institutional Research Ethics Regulatory Committee, of the North-West University, South Africa, was obtained as well as the consent of the participants which was verbally requested prior to the interview. Verbal consent from parents or guardians of participants less than 16 years old was also sought prior to seeking consent from individual participants.

### Statistical approach

Both the male and female data are analysed in the present study for youth aged 15–34 years. A total of 2806 and 2771 of women and men respectively were used for the quantitative component of the study. To ensure representativeness, as well as to correct for non-response, the data is weighted taking into consideration the complex survey design, using the ‘svyset’ command in Stata. The dependent variable for the analysis is water consumption which is measured as the number of glasses of water an individual consumes in a day (‘How many glasses of water do you drink in one day on average?’). The distribution of the variable for females ranged from ‘0’ to ‘20’ glasses; the distribution for males ranged from ‘0’ to ‘15’ glasses of water. Thus, the dependent variable was treated as a count response and thus lent itself to analysis using the Poisson regression model.

### Qualitative approach

The qualitative data analysis involved the coding of the transcribed (textual) data. The textual data was then analysed using the Atlas Ti software through development of descriptive codes and analytical themes from the transcribed data. To obtain views collectively, perceptions were looked at across the whole data set and quotations from the interviews were used to substantiate claims.

## Results

### Quantitative data

Table 1 shows the distribution of the sample characteristics. The table shows that most respondents in the sample are in the age group 15–24 years. Specifically, among females, the age group 15–24 years comprises 55% of females, and among male youth, most (58%) belong to the age group 15–24. From the distribution, female youth with a secondary education are the largest with a proportion of 61%, while only 4% reported higher level of education. Close to two thirds (65%) of males also have secondary level of education, while fewer (8%) males reported the attainment of a higher level of education. More than three quarters (79%) of female youth are Christian, while about 3% belong to the traditional/spiritualist or ‘other’ religion.

**Table 1:** Distribution of sample characteristics of respondents

Variables	Female		Male	
Age	No.	Percentage	No.	Percentage
15–24	1553	55.33	1615	58.27
25–34	1253	44.67	1156	41.73
Educational level				
No education	450	15.35	276	9.97
Primary	541	19.30	460	16.62
Secondary	1708	60.86	1806	65.15
Higher	126	4.49	229	8.26
Religion				
Christians	2215	78.95	2062	74.41
Muslims	424	15.13	452	16.29
Traditional/spiritualists	86	3.05	127	4.60
Other	81	2.87	130	4.69
Ethnicity				
Akan	1424	50.76	1320	47.64
Ga/Dangme	206	7.37	166	6.00
Ewe	349	12.45	401	14.48
Mole-Dagbani	428	15.25	456	16.47
Other	397	14.17	427	15.41
Marital status				
Never married	1349	48.07	1875	67.67
Married/living together	1308	46.62	821	29.61
Formerly married	149	5.31	75	2.73
Place of residence				
Urban	1463	52.12	1326	47.87
Rural	1343	47.88	1445	52.13
Region of residence				
Western	237	8.47	251	9.05
Central	225	8.04	227	8.20
Greater Accra	534	19.04	446	16.09
Volta	227	8.11	256	9.25
Eastern	281	10.04	264	9.53
Ashanti	590	21.03	554	20.00
Brong-Ahafo	262	9.35	252	9.09
Northern	249	8.90	292	10.54
Upper East	126	4.50	146	5.26
Upper West	70	2.51	83	3.01
Total	2806	100.00	2771	100.00
Water intake (ranging from 0 to 20 glasses for females and 0–15 glasses for males	Mean	SD	Mean	SD
	4.99602	2.202203	5.76932	2.248927

Source: computed from the GDHS 2008 data file

Among male youth, most (74%) are Christian, followed by Muslims (16%). In terms of ethnicity, two quarters (50%) of females are Akan, and the Ga-Dangmes constitute the least proportion (7%). A higher proportion of male youth are Akan (47%), followed by the Mole-Dagbani group; the least proportion of male youth belongs to the Ga-Dangme. Regarding marital status, more than two fifths (48%) of females are single, with only 5% who are formerly married. Male youth have about two thirds (67%) singles, followed by those who are married/living together; the formerly married males constitute the least proportion in the male sample.

According to Table 1, half of the female respondents are urban dwellers. Male residents in the rural areas constitute a higher proportion (52%) of male youth. Most females reside in the Ashanti region followed by the Greater Accra region (21 and 19% respectively). One fifth (20%) of male youth live in the Ashanti region. This is followed by the Greater/Accra region with 16% of male respondents. In general, the Upper East and Upper West regions have the least proportions of youths in the sample. The mean water intake for females is 4.99 (SD = 2.202) and 5.76932 (SD = 2.248) among male youth. On average, females and males approximately consume five and six glasses of water respectively.

Tables 2, 3, 4, 5 and 6 show results of the *t* test, one-way ANOVA and Tukey’s post hoc test for both male and female youth. Table 2 shows the results of the independent *t* test. The *t* test results for females show statistically significant differences in the means for age (*p* < 0.001) but not for place of residence (*p* = 0.0650) as indicated by the *p* values. However, for males, the *t* test has shown statistically significant differences in the means for age (*p* < 0.001) as well as place of residence (*p* < 0.001). The ANOVA results for female youth presented in Table 3 show statistically significant differences between the groups, ethnicity, marital status and region of residence while no differences are observed for education and religion.

**Table 2:** Mean differences between groups

Independent *t* test result						
Variable (group)	Males		*p* value	Females		*p* value
Age	Mean	SD	0.0000	Mean	SD	0.0000
15–24	5.429612	2.222018		4.673052	2.113792	
25–34	6.110424	2.25226		5.318817	2.254071	
Place of residence			0.0000			0.0650
Urban	5.917978	2.238825		5.036309	2.161226	
Rural	5.544819	2.26154		4.882272	2.23143	

*p* value is significant at < 0.05

Source: computed from GDHS 2008 data file

**Table 3:** One-way ANOVA results of females

	SS	df	MS	*F*	*p* value
Educational level					
Between groups	24.3146076	3	8.1048619	1.68	0.1698
Within groups	13,432.1604	2779	4.83345103		
Religion					
Between groups	22.1531784	3	7.38439281	1.53	0.2053
Within groups	13,434.3218	2779	4.8342288		
Ethnicity					
Between groups	88.6201007	4	22.1550252	4.60	0.0010
Within groups	13,367.8549	2778	4.81204281		
Marital status					
Between groups	149.618994	2	74.8094969	15.63	0.0000
Within groups	13,306.856	2780	4.78663886		
Region					
Between groups	421.360972	9	46.8178858	9.96	0.0000
Within groups	13,035.1141	2773	4.70072631		

*p* value is significant at < 0.05

Source: computed from GDHS 2008 data file

**Table 4:** One-way ANOVA results of males

	SS	df	MS	F	*p* value
Educational level					
Between groups	80.689389	3	26.896463	5.30	0.0012
Within groups	14,099.3808	2776	5.07902765		
Religion					
Between groups	39.6559646	3	13.2186549	2.60	0.0509
Within groups	14,140.4142	2776	5.09380914		
Ethnicity					
Between groups	140.26961	4	35.0674024	6.93	0.0000
Within groups	14,039.8005	2775	5.05938758		
Marital status					
Between groups	151.142894	2	75.5714472	14.96	0.0000
Within groups	14,028.9272	2777	5.05182832		
Region					
Between groups	599.51824	9	66.6131378	13.59	0.0000
Within groups	13,580.5519	2770	4.90272632		

*p* value is significant at < 0.05

Source: computed from GDHS 2008 data file

**Table 5:** Pairwise results of socio-demographic characteristics

Tukey’s post hoc test results of females			
Water consumption	Contrast (mean)	Standard error	*p* value
Ethnicity			
Mole-Dagbani vs Ewe	− 0.4110991	0.1489361	< 0.05
Other vs Ewe	− 0.6350575	0.1554869	< 0.05
Marital status			
Married vs never married	0.4737177	0.085051	< 0.05
Region			
Upper East vs Western	1.115569	0.2120391	< 0.05
Northern vs Central	− 0.138987	0.2086699	< 0.05
Eastern vs Greater Accra	− 0.1392733	0.1668875	< 0.05
Northern vs Greater Accra	− 0.3846678	0.1678195	< 0.05
Upper East vs Greater Accra	0.7525936	0.1883944	< 0.05
Eastern vs Volta	− 0.3709831	0.1950631	< 0.05
Ashanti vs Volta	− 0.7400901	0.1760586	< 0.05
Upper East vs Volta	0.5208837	0.2137536	< 0.05
Northern vs Eastern	− 0.2453945	0.1850735	< 0.05
Upper East vs Eastern	0.8918669	0.2039148	< 0.05
Upper West vs Eastern	− 0.7275843	0.1877788	< 0.05
Upper East vs Ashanti	1.260974	0.1858179	< 0.05
Upper East vs Brong Ahafo	1.209615	0.2089077	< 0.05
Upper East vs Northern	1.137261	0.2046783	< 0.05
Upper West vs Upper East	− 1.619451	0.2071277	< 0.05

Only significant pairings are shown in the table

Source: computed from GDHS 2008 data file

**Table 6:** Pairwise results of socio-demographic characteristics

Tukey’s post hoc test results for males			
Water consumption	Contrast (mean)	Standard error	*p* value
Educational level			
Higher vs no education	0.5281515	0.196671	< 0.05
Higher vs primary	0.7166027	0.1841116	< 0.05
Higher vs secondary	0.4391311	0.1640866	< 0.05
Ethnicity			
Ewe vs Akan	0.3926644	0.1326872	< 0.05
Mole-Dagbani vs Akan	− 0.3427258	0.1093261	< 0.05
Mole-Dagbani vs Ewe	− 0.7353902	0.1429182	< 0.05
Marital status			
Married vs never married	0.4930049	0.0936365	< 0.05
Region			
Greater Accra vs Western	0.9992054	0.1861614	< 0.05
Volta vs Western	0.8639772	0.2013107	< 0.05
Brong-Ahafo vs Central	− 0.8578511	0.221852	< 0.05
Upper West vs Central	− 0.7571981	0.2106115	< 0.05
Eastern vs Greater Accra	− 0.8724636	0.1805087	< 0.05
Ashanti vs Greater Accra	− 0.8125827	0.1570069	< 0.05
Brong-Ahafo vs Greater Accra	− 1.479911	0.1856714	< 0.05
Northern vs Greater Accra	− 1.089977	0.1701384	< 0.05
Upper East vs Greater Accra	− 0.7099007	0.1914884	< 0.05
Upper West vs Greater Accra	− 1.379258	0.1720835	< 0.05
Eastern vs Volta	− 0.7372355	0.1960952	< 0.05
Ashanti vs Volta	− 0.6773545	0.174703	< 0.05
Brong-Ahafo vs Volta	− 1.344683	0.2008577	< 0.05
Northern vs Volta	− 0.9547493	0.1865932	< 0.05
Upper West vs Volta	− 1.24403	0.1883685	< 0.05
Brong-Ahafo vs Ashanti	− 0.667328	0.1796129	< 0.05
Upper West vs Ashanti	− 0.566675	0.1655285	< 0.05
Upper East vs Brong-Ahafo	0.77001	0.210422	< 0.05
Upper West vs Upper East	−0.669357	0.1985353	< 0.05

Only significant pairings are shown in the table

Source: computed from GDHS 2008 data file

On the other hand, the ANOVA results presented in Table 4 for males show statistically significant differences for educational level, ethnicity, marital status and region of residence but no statistically significant differences in the means are observed for religion.

Following the ANOVA test for females which showed that the means for water intake were not all equal for ethnic group, marital status and region of residence, the Tukey’s post hoc test to determine which means were different was done, and the results are presented in Table 5. In terms of ethnicity, the results show that females from the Mole-Dagbani group consume more water than those from the Ewe group (*p* = 0.046) and females belonging to the ‘other’ ethnic group consume more water than Ewe females (*p* < 0.001).

Regarding marital status, more water consumption is significantly associated with the married group than the never married group (*p* < 0.001). Also, more water consumption is associated with females residing in the Upper East region compared to those residing in the Western region (*p* < 0.001), and females in the Upper East region compared to females in the Central region consume more water (*p* < 0.001).

The post hoc test (see Table 6) for male youth also shows more water consumption among males in the higher education group than the no education group (*p* = 0.037); males with higher education consume more water compared to those with primary education (*p* < 0.001), and males with higher education take in more water than males with secondary level of education (*p* = 0.038). Regarding ethnicity, males in the Ewe group are found to significantly consume more water than males in the Akan group (*p* = 0.026); males belonging to the Mole-Dagbani group compared to those in the Akan group drink more water (*p* = 0.015), and more water consumption is significantly associated with males in the Mole-Dagbani group compared to their Ewe counterparts (*p* < 0.001).

In terms of marital status, more water consumption is significantly observed in married males as against their never married counterparts (*p* < 0.001). With regard to region of residence, differences in water consumption are observed between several comparisons, for example, males residing in the Greater Accra region drink more water than males in the Western region (*p* < 0.001); also, males in the Volta region consume more water than those in the Western region (*p* < 0.001), and male residents in the Brong/Ahafo region drink more water compared to their counterparts in the Central region (*p* = 0.004).

To examine the predictors of water consumption, the Poisson regression technique is employed. Table 7 shows that age and region of residence are significantly associated with water intake among male youth, while age, ethnicity, marital status and region of residence are associated with water intake among females.

**Table 7:** Poisson regression analysis of the association between socio-demographic characteristics and water intake

	Male				Female			
Variable	IRR	S.E	95%	CI	IRR	S.E	95%	CI
Age								
15–24(RC)				1.000				1.000
25–34	1.112*	0.022	1.070	1.156	1.103*	0.025	1.054	1.153
Level of education								
No education(RC)				1.000				1.000
Primary	0.974	0.030	0.917	1.035	0.982	0.033	0.919	1.051
Secondary	1.008	0.028	0.954	1.064	1.023	0.031	0.964	1.085
Higher	1.021	0.039	0.948	1.100	1.027	0.049	0.934	1.129
Religion								
Christian(RC)				1.000				1.000
Moslems	1.016	0.028	0.961	1.073	1.011	0.038	0.940	1.088
Traditional/spiritualists	1.053	0.045	0.968	1.147	1.038	0.052	0.941	1.145
Other	1.062	0.042	0.982	1.149	0.995	0.054	0.894	1.106
Ethnicity								
Akan	1.052	0.042	0.972	1.139	1.022	0.041	0.943	1.107
Ga-Dangme (RC)				1.000				1.000
Ewe	1.022	0.044	0.938	1.114	1.027	0.049	0.934	1.129
Mole-Dagbani	1.024	0.054	0.922	1.136	0.927	0.053	0.828	1.038
Other	1.051	0.048	0.960	1.149	0.919**	0.045	0.834	1.013
Marital status								
Never married (RC)				1.000				1.000
Married/living together	1.024	0.020	0.984	1.065	1.051**	0.027	0.999	1.106
Formerly married	0.985	0.053	0.886	1.095	1.017	0.047	0.929	1.115
Place of residence								
Urban	1.028	0.023	0.983	1.075	1.044	0.030	0.986	1.106
Rural (RC)				1.000				1.000
Region of residence								
Western	0.855*	0.040	0.780	0.937	0.962	0.049	0.869	1.064
Central	0.910*	0.040	0.834	0.993	0.971	0.050	0.877	1.075
Greater Accra (RC)				1.000				1.000
Volta	1.019	0.041	0.941	1.103	1.053	0.053	0.954	1.162
Eastern	0.892*	0.040	0.816	0.974	0.988	0.039	0.914	1.069
Ashanti	0.890*	0.029	0.835	0.949	0.907*	0.033	0.844	0.975
Brong Ahafo	0.773*	0.029	0.719	0.832	0.956	0.056	0.852	1.073
Northern	0.851*	0.035	0.785	0.922	0.985	0.062	0.871	1.114
Upper East	0.922	0.045	0.837	1.016	1.258*	0.069	1.130	1.400
Upper West	0.823*	0.038	0.751	0.902	0.923	0.051	0.828	1.029
	_hatsq = 0.551				_hatsq = 0.584			

Source: computed from GDHS 2008 data file

*< 0.05, **< 0.10

There is a significant association between age and water intake. Specifically, male and female youth aged 25–34 would be expected to consume 11.2 and 10.3% more glasses of water respectively compared to their counterparts aged 15–24 years. Ethnicity is predictive of water intake among the youth in Ghana. For example, females in the ‘other’ ethnic category compared to Ga-Dangme females, would be expected to consume 8.1% less glasses of water. Marital status in females is associated with water intake. Females who are married/living together are expected to consume 5.1% more glasses of water compared to those never married.

Youth in most regions consume less glasses of water compared to youth in the Greater Accra region. Males residing in the Western, Central, Eastern, Ashanti, Brong/Ahafo, Northern and Upper West regions would be expected to consume 14.5, 9, 10.8, 11, 22.7, 14.9 and 17.7% less glasses of water respectively compared to their counterparts in the Greater Accra region. And for female youth, the water consumption of those in the Ashanti region would be expected to decrease by 9.3% glasses compared to those in the Greater Accra region. However, females residing in the Upper East region would be expected to consume 25.8% more glasses of water than their counterparts in the Greater Accra region.

### Qualitative data

As the quantitative analysis of water consumption above show, most youth do not take in eight or more glasses of water (85 and 72% in females and males respectively) as recommended in the RHNP by the MoH of Ghana. As a result of this inadequate intake of water by the youth, the qualitative data (focus group discussions) was used to ascertain the reasons why the youth do not consume enough water by assessing the importance they attach to the consumption of water, how common it is among them and perceived barriers to its adequate consumption. The discussions revolved around the following themes:

#### Importance attached to water consumption

Knowing the importance the youth attach to water consumption provides knowledge about why they may or may not consume the recommended amounts.

##### Therapeutic effect

Most participants agreed that drinking water has a therapeutic effect on the human body. In fact, almost all of the participants agreed that we consume water for the health benefits which include reducing risk to obesity and NCDs as illustrated by the following quotes:

A few of my age mates know that health wise it is good to drink a lot of water because these days when you go to the net [internet] you will see all kinds of therapies that are talking of water, how it helps clean the body, prevent heart attack and all those things.

(Female, 15–24 years, English)

Water is life. Water is important and you need it. I like taking water because it clears your system and makes the blood flow well. It does not make you to dehydrate.

(Female, 15–24 years, Ga)

##### Social status

But the youth may consume water not because of its health benefits but because of the status it appears to confer on such behaviour and concerns about its safety. There appears to be increasing concern in the Ghanaian society about the cleanliness of water, especially drinking water outside the home. This concern has led to the commercialisation of drinking water in the form of bottled and sachet water in the society, especially in urban areas. Thus, though the properties of water do not change, the brand of water consumed signifies ones’ economic standing, especially buying bottled water brands. On the other hand, the commercialisation of water to address its safety has affected affordability and hence the inadequate intake of water by the youth.

I think if you can afford brand name bottled water or let’s say you have a dispenser at home, like you are considered rich. Though generally water is water, but the brand of water you take that is what gives you the status.

(Male, 15–24 years, English)

But adequate water intake was noted to be uncommon among the youth because of the availability of fizzy drinks, tea, etc. as a result of the modernisation process. This explains why adequate water intake is uncommon among the youth as shown in the following quote:

No, mineral drinks are now in the system and not expensive so people buying them. This place has turned out to be a place for selling tea and people buy it. Someone said to me that coke is his water after eating food for him to be satisfied.

(Female, 15–24 years, Ga)

#### Perceived barriers to water consumption

Even though the youth know the importance of water consumption, the data clearly shows that they do not consume adequate amount of water. The informants mentioned as barriers associated with inadequate water consumption are physiological factors (thirst, urination and hunger), accessibility, external factors (e.g. weather) and personal factors (e.g. weight maintenance).

##### Accessibility

But it is rather ironical that this emerging trend of the youth consuming water as a status symbol creates an unintended problem of accessibility in a context where most people are poor and therefore can hardly afford the spiralling cost of bottled water. Thus, cost associated with the commercialization of water in the society has been identified as a barrier preventing adequate water intake among the youth. This is illustrated in the following quotes:

For me, what will prevent me from taking water is that maybe the water is not there.

(Male, 15–24 years, English)

Also our choice of water. Some of us prefer our water cold so, if where I am I don’t have any fridge and I don’t have money to get a cold water, I’ll wait when I get to the house where there is water in the fridge I’ll drink water.

(Female, 15–24 years, English)

Then it is not advisable to drink any public water anywhere. So we prefer the sachet and the bottled water. So if you don’t have the money to afford, you are not taking the water at all.

(Female, 15–24 years, English)

##### Physiological factors

The physiological barriers relate to body functioning. In other words, it appears that like most people in the country, the water intake by the youth is for the physiological need to quench their thirst either after eating a meal, when they feel dehydrated or for others as a result of frequency of urination resulting from adequate water intake. Thus, the ability to consume water adequately depends on these physiological demands of the body as illustrated by the following quotes:

Also it is not frequent that you feel thirsty, and then if you don’t feel thirsty you won’t take water.

(Female, 15–24 years, English)

They are not thirsty and I don’t take water that much because I will be urinating a lot.

(Female, 25–34 years, Ga)

You know mostly when you eat that is when you take in water. Me when I eat I’m able to take in more water but if I don’t eat I’m not able to take water.

(Female, 25–34 years, English)

You see you usually take water when you are eating.

(Male, 25–34 years, English).

##### Weather

Ghana is geographically located in a tropical climate, which requires adequate intake of water to hydrate the body. However, according to the youth the intake of water depends on the prevailing weather as well as access to potable water as illustrated by the following quote:

Sometimes it depends on the weather. When the weather is dry you take much but if the weather is cold the amount of water you take will reduce…

(Male, 15–24 years, English)

##### Weight maintenance

There is a misinformation in regard to what water does for the body. It is perceived as a means of becoming overweight. Consequently, some youth are reluctant to drink adequate amount of water indicating the lack of education on the health benefits of adequate water intake as illustrated below:

Some also have the perception that when they take in more water, they grow fat. Because of that they reduce the water intake. And that has been their reason for doing that.

(Female, 25–34 years, Ga).

## Discussion

In the absence of any empirical study on the relationship between socio-demographic factors and water intake among the youth in Ghana, the present study sought to examine this relationship using the 2008 Demographic and Health Survey data. It was found that on the whole, about 85 and 72% of females and males respectively consume less than eight glasses of water although water consumption was identified to be important. Moreover, the study found that among males, age and region of residence were the significant factors, while age, ethnicity, marital status and region of residence were found to be the most significant among females. Age of individuals has been identified to determine their actions and inactions [16]. The current finding about the positive influence of age on water intake among the youth (male and female) is similar to findings reported by Park et al. [14] who found younger respondents (≤ 15 years) consumed less water.

However, other studies have found that older age individuals consumed less water than younger people [12, 13]. As far as the finding in Ghana by the present study is concerned, older youth consume more water probably because of the type of occupation (e.g. farming) they usually engage in. As the data showed, most youth consume water to meet such physiological need as thirst, a need that may be triggered by such physical activity as farming.

In terms of ethnicity, females belonging to the ‘other’ ethnic group consume less water than youth in all other ethnic groups. This finding is corroborated by Goodman et al. [13] in the USA who found a low probability for subjects belonging to ‘other’ ethnic group in drinking adequate amount of water compared to whites, although other studies [12, 14] found no association between ethnicity and water intake. As far as the finding of the association between ethnicity and water consumption in Ghana is concerned, the variation in water consumption is probably due to the relative access to and or availability of water to members of the ‘other’ ethnic group as a result of the commercialization of potable water.

With regard to marital status, females who are married/living together consumed more water than youth in all other marital statuses. This may be part of the general advantage marriage confers as opposed to being single, divorced or widowed. The chances of married couples sharing information about such healthy behaviours as water consumption are higher than among persons who are not married.

Similarly, the finding about the relationship between region of residence and water consumption is consistent with the study by Goodman et al. [13] who found among Americans that there was an association between region of residence and the consumption of water. Even though Ghana is generally a warm country, regions in the northern parts of the country are considerably warmer and dryer than those in the southern parts such as the Greater Accra region. However, the consumption of water depends largely on the availability of and access to potable water which also differs between and within regions.

The present study has shown that the youth are aware of both the therapeutic effect of and the social status water consumption confers on a person. However, factors such as thirst, frequent urination and hunger and physical access and cost act as barriers to its frequent intake by the youth.

This study examined factors relating to water intake among young Ghanaians 15–34 years old. However, caution must be exercised in interpreting the qualitative results of this study. Thus, though the findings may shed light to the present situation, they may not be generalised to all young people in Ghana. Secondly, findings were based on respondents’ self-reports and may therefore be influenced with errors associated with memory lapse and social desirability.

## Conclusion

In conclusion, the study has demonstrated that both individual-level (e.g. age, ethnicity, marital status) and contextual factors (urbanisation and region) affect the intake of water among the youth in Ghana. Thus, in terms of policy intervention, attempts at improving water intake to reduce the risk to obesity and consequently to non-communicable diseases among young Ghanaians should consider these socio-demographic characteristics and barriers identified from the FGDs.

## Abbreviations

FGDs: Focus group discussions
GDHS: Ghana Demographic and Health Survey
MoH: Ministry of Health
NCDs: Non-communicable diseases
RHNP: Regenerative Health and Nutrition Programme
WHO: World Health Organization
